# Interaction Between Macrophage Migration Inhibitory Factor and CD74 in Human Immunodeficiency Virus Type I Infected Primary Monocyte-Derived Macrophages Triggers the Production of Proinflammatory Mediators and Enhances Infection of Unactivated CD4^+^ T Cells

**DOI:** 10.3389/fimmu.2018.01494

**Published:** 2018-06-27

**Authors:** César Trifone, Jimena Salido, María Julia Ruiz, Lin Leng, María Florencia Quiroga, Horacio Salomón, Richard Bucala, Yanina Ghiglione, Gabriela Turk

**Affiliations:** ^1^CONICET-Universidad de Buenos Aires, Instituto de Investigaciones Biomédicas en Retrovirus y Sida (INBIRS), Buenos Aires, Argentina; ^2^Department of Medicine, Yale University School of Medicine, New Haven, CT, United States

**Keywords:** human immunodeficiency virus, CD74, macrophage migration inhibitory factor, primary monocyte-derived macrophages, CD4^+^ T-cells, immunopathogenesis

## Abstract

Understanding the mechanisms of human immunodeficiency virus type I (HIV-1) pathogenesis would facilitate the identification of new therapeutic targets to control the infection in face of current antiretroviral therapy limitations. CD74 membrane expression is upregulated in HIV-1-infected cells and the magnitude of its modulation correlates with immune hyperactivation in HIV-infected individuals. In addition, plasma level of the CD74 activating ligand macrophage migration inhibitory factor (MIF) is increased in infected subjects. However, the role played by MIF/CD74 interaction in HIV pathogenesis remains unexplored. Here, we studied the effect of MIF/CD74 interaction on primary HIV-infected monocyte-derived macrophages (MDMs) and its implications for HIV immunopathogenesis. Confocal immunofluorescence analysis of CD74 and CD44 (the MIF signal transduction co-receptor) expression indicated that both molecules colocalized at the plasma membrane specifically in wild-type HIV-infected MDMs. Treatment of infected MDMs with MIF resulted in an MIF-dependent increase in TLR4 expression. Similarly, there was a dose-dependent increase in the production of IL-6, IL-8, TNFα, IL-1β, and sICAM compared to the no-MIF condition, specifically from infected MDMs. Importantly, the effect observed on IL-6, IL-8, TNFα, and IL-1β was abrogated by impeding MIF interaction with CD74. Moreover, the use of a neutralizing αMIF antibody or an MIF antagonist reverted these effects, supporting the specificity of the results. Treatment of unactivated CD4^+^ T-cells with MIF-treated HIV-infected MDM-derived culture supernatants led to enhanced permissiveness to HIV-1 infection. This effect was lost when CD4^+^ T-cells were treated with supernatants derived from infected MDMs in which CD74/MIF interaction had been blocked. Moreover, the enhanced permissiveness of unactivated CD4^+^ T-cells was recapitulated by exogenous addition of IL-6, IL-8, IL-1β, and TNFα, or abrogated by neutralizing its biological activity using specific antibodies. Results obtained with BAL and NL4-3 HIV laboratory strains were reproduced using transmitted/founder primary isolates. This evidence indicated that MIF/CD74 interaction resulted in a higher production of proinflammatory cytokines from HIV-infected MDMs. This caused the generation of an inflammatory microenvironment which predisposed unactivated CD4^+^ T-cells to HIV-1 infection, which might contribute to viral spreading and reservoir seeding. Overall, these results support a novel role of the MIF/CD74 axis in HIV pathogenesis that deserves further investigation.

## Introduction

The pandemic of human immunodeficiency virus/acquired immunodeficiency syndrome (HIV/AIDS) is still a major public health concern worldwide. Combined antiretroviral therapy (cART) can diminish the viral load (VL) to undetectable levels, reducing not only morbidity and mortality but also transmission risks, with the subsequent impact on the dynamic of the global epidemic (1). However, cART has several limitations like the need of daily doses, the development of viral resistance, and toxicity. More importantly, the rebound of VL levels in patients who discontinue cART suggests the presence of long-lived viral reservoirs that are resistant to cART, hampering the cure of the infection. In addition, it is being increasingly clear that even effectively treated HIV-infected individuals have a greater risk of experiencing non-AIDS related morbidity and mortality events than age-matched HIV-uninfected adults, indicating that even effective cART cannot fully restore health. Most of these complications are related to immune dysfunction and inflammation and include gut-associated mucosal disruption, lymphoid tissue damage, liver dysfunction, and monocyte/macrophage activation which ultimately lead to the development of coagulopathies, atherosclerosis, vascular dysfunction, and frailty, among other effects (2). Thus, understanding the mechanisms underlying HIV persistence and irreversible immune damage is extremely important to fight the infection and its consequences.

CD4^+^ T-cells are the major targets of HIV infection followed by macrophages. Productive viral replication is supported mostly in activated CD4^+^ T-cells, which culminates in cell apoptosis. Conversely, macrophages are less permissive to HIV-1 infection albeit more resistant to virus-mediated cell killing, thus viral replication proceeds for a longer time compared to T cells (3, 4). Both cell types play an important role since the onset of infection to the development of chronic inflammation regardless of the different viral replication strategies maintained in each cell type.

CD74 (also known as invariant chain or Ii) is a non-polymorphic type II integral membrane protein expressed by antigen-presenting cells. It was first described to act as a major histocompatibility class II-associated chaperone; but now, it is increasingly understood as a versatile protein with multiple roles (5–7). In the context of HIV-1 infection, surface CD74 expression is upregulated by Nef (8, 9) and Vpu (10) viral proteins. Moreover, accumulated data suggest that Nef-mediated CD74 upregulation might play an important role in HIV immunopathogenesis as: (i) this activity is conserved among *nef* alleles from HIV-1 primary isolates, HIV-2, and SIV (9, 11) as well as HIV-1 BF inter-subtype recombinant forms (12), (ii) it has been documented in *in vitro* infected cell lines [HeLa-CIITA, MelJuSo, and THP-1 (8, 13)] and also in primary CD4^+^ T-cells and monocyte-derived macrophages (MDMs) (13, 14), and (iii) modulation levels differ among progressive versus non-progressive infected individuals, both in adult (9) and pediatric populations (13). Moreover, our group has demonstrated that CD74 upregulation occurs on naturally infected MDMs obtained directly from HIV^+^ subjects and that the magnitude of this upregulation correlates with the level of immune activation in those subjects, providing evidence for the contribution of the HIV-mediated CD74 upregulation to immune damage during the course of infection (15).

One of the alternative activities described for CD74 is its ability to serve as the high-affinity binding component of the heteromeric receptor for macrophage migration inhibitory factor (MIF) (16–18). MIF is a proinflammatory cytokine that plays a key role in anti-stress and anti-microbial responses. It is secreted by different immune cells including T and B lymphocytes, macrophages, monocytes, and dendritic cells among others (19). MIF has been related to the pathogenesis of diverse inflammatory, infectious, autoimmune, and metabolic diseases as well as different types of cancer (20–33). During HIV infection, increased MIF plasma levels have been observed during the acute phase of infection and remained elevated (34, 35). On the other hand, it has been demonstrated that MIF was heavily produced by *in vitro* infected peripheral blood mononuclear cells (PBMCs) and also by uninfected gp120-stimulated PBMCs. Moreover, the addition of exogenous recombinant MIF to *in vitro* infected PBMCs increased viral replication (34).

Despite the fact that MIF is a key component of the inflammatory immune response, that it is elevated in plasma from HIV-infected subjects, and that the virus itself modulates the surface expression of its receptor, no reports have explored the role of the MIF/CD74 axis in HIV immunopathogenesis. Thus, the aim of this work was to study the effect of MIF/CD74 interaction on the phenotype and the function of primary HIV-infected MDMs, and how this axis determines the environment to modulate CD4^+^ T-cell permissiveness to infection.

## Materials and Methods

### Primary Human MDM and CD4^+^ T-Cell Purification and Culture

Buffy coats from healthy donors were used to obtain PBMCs by Ficoll-Hypaque (GE Healthcare Life Sciences, USA) density gradient centrifugation. Monocytes were then separated from PBMCs by Percoll (GE Healthcare Life Sciences, USA) gradient technique. Isolated monocytes (purity >80% measured by flow cytometry) were further purified by adherence to plastic plates in RPMI 1640 medium (HyClone, GE Healthcare Life Sciences, USA). Non-adherent cells were removed after 2 h plating by means of extensive washes. Adherent cells were allowed to differentiate into MDMs in RPMI 1640 medium supplemented with 10% fetal bovine serum (FBS, Gibco, Thermo Fischer Corporation, USA), 2 mM l-glutamine (Sigma-Aldrich, USA), 100 U/ml penicillin (Sigma-Aldrich USA), 100 µg/ml streptomycin (Sigma-Aldrich, USA), and 10 mM HEPES (Sigma-Aldrich) (from now on complete RPMI medium) plus 20 ng/ml recombinant granulocyte monocyte-colony stimulating factor (GM-CSF, Miltenyi, Germany) for 4 days. After differentiation, MDM purity was analyzed by flow cytometry and only donors with >90% purity were used in subsequent assays.

CD4^+^ T-cells were isolated from buffy coats by negative selection using the RosetteSep kit (Stem cell, Canada). Purified cells (>95% purity by flow cytometry) were cultured in complete RPMI medium plus 25 ng/ml IL-2 (BioLegend, USA). Culture plates were incubated at 37°C in a humidified atmosphere with 5% CO_2_.

### Virus Production and Infections

GFP-expressing X4-tropic HIV-1 virus stock was produced by transfecting 293 T cells using the X-tremeGENE 9 DNA transfection reagent (Roche, Switzerland) with the pBR43IeG-nef^+^plasmid (kindly provided by Dr. Michael Schindler). This plasmid encodes the full-length HIV genome plus the reporter protein GFP (pBR-NL4-3 nef-IRES-eGFP, NefWT virus). Similarly, a Nef-defective virus (ΔNef) was produced using the pBR43IeG-nefSTOP plasmid. When stated, a pseudotyped X4-tropic virus, generated by adding a plasmid encoding the vesicular stomatitis virus (VSV) protein G to the transfection solution, was used. Also, an R5-tropic HIV-1 viral stock was produced by infecting primary MDMs from healthy donors with the HIV-1 BAL strain. Finally, an R5- and a dual (R5X4)-tropic transmitted/founder (T/F) infectious molecular clones (IMCs) were selected from the full panel of T/F IMCs available at the NIH AIDS Reagent program [Division of AIDS, NIAID, NIH: Cat #11746 and 11744, respectively, from Dr. John Kappes (36–39)]. Both T/F viral stocks were produced by transfecting 293 T cells using the X-tremeGENE 9 DNA transfection reagent. Culture supernatants were harvested 48 h post-transfection (for the NL4-3 and T/F viruses) or 14 days post-infection (for the BAL R5-tropic stock). In all cases, culture supernatants were clarified by centrifugation at 600 *g* for 15 min at 4°C, fractioned and stored at −80°C until use. Viral titer was estimated by p24 antigen quantitation by ELISA (Sino Biological Inc., China).

Monocyte-derived macrophages were infected with the R5-tropic viruses (either with the BAL or the R5-tropic T/F strain) using a ratio of 1 ng p24/10^6^ cells. MDMs to be evaluated by immunofluorescence microscopy were infected with the VSV-G pseudotyped X4-virus and CD4^+^ T-cells were infected using the X4-tropic virus (either the NL4-3 or the dual-tropic T/F strain) by spinoculation (1,200 *g* for 1.5 h at 37°C) using a ratio of 150 ng p24/10^6^ cells in both cases. After adsorption, the inoculum was removed and cells were washed twice in RPMI medium.

### Human Samples

Plasma from 13 HIV seronegative healthy donors (HIV−) and 13 individuals with recent HIV-1 infection (HIV+) were obtained. Samples from HD were obtained from eligible voluntary blood donors >18 years old who completed a survey on blood donation which particularly excludes persons who had been exposed to HIV; and were screened for serological markers before being accepted as donors. HIV-infected subjects were enrolled as part of an ongoing acute/early primary HIV infection cohort from Argentina (40–45). This study was reviewed and approved by two institutional review boards: *Comité de Ética Humana, Facultad de Medicina, Universidad de Buenos Aires* and *Comité de Bioética, Fundación Huésped (Buenos Aires, Argentina)*. Both HIV-infected participants and HD provided written informed consents accepting to participate in this study in accordance with the Declaration of Helsinki.

### Immunofluorescence Microscopy

Monocyte-derived macrophages obtained as mentioned above were cultured over coverslips and infected either with the pseudotyped GFP-expressing Nef wild type (WT) virus or the pseudotyped GFP-expressing Nef-defective (ΔNef) virus. Uninfected cells were used as controls. Three days post-infection, MDMs were fixed and permeabilized with Cytofix-Cytoperm buffer (BD Biosciences) following the manufacturer’s instructions and blocked with Cytoperm wash buffer plus 2% FBS. MDMs were subsequently stained overnight with an anti-CD44 antibody (BioLegend). The following day, cells were washed three times and stained with an Alexa546-conjugated anti-mouse antibody (Jackson, MS, USA) during 1 h. Finally, cells were washed three times, treated again with cytofix-cytoperm buffer, blocked, and stained overnight with an APC-conjugated anti-CD74 antibody (BioLegend, USA). After three final washes, cells were fixed and mounted with DAPI-Fluoromount-G (Thermo Fisher Scientific) and analyzed in an Olympus FV-1000 (Olympus, Tokyo, Japan) microscope with a Plapon 60×/1.42 NA oil immersion objective and using FV10-ASW v.01.07.03.00 software. Cross-sectional quantitation of mean fluorescence intensity (mFI) was performed using Image J software. Single stained controls were performed in order to exclude channel spillover (cells stained only with APC-conjugated anti-CD74 antibody or the anti-CD44 antibody followed by Alexa546-conjugated anti-mouse antibody staining). Also, individual isotype controls were performed in order to exclude unspecific antibody binding and cross-reactivity with the secondary antibody.

### Evaluation of CD74 Modulation

After infection with the WT or ΔNef GFP-expressing viruses, MDMs were detached with trypsin (Gibco) and stained with an anti-CD74-PE (Santa Cruz Biotechnology, USA). Cells were washed and analyzed in a BD FACSCanto flow cytometer (BD Biosciences) using the FACSDiva v8.0.1 software (BD Biosciences) or FlowJO v10 (Data Analysis Software, LLC). HIV-mediated CD74 upregulation was calculated as the ratio between the FL2 median fluorescence intensity (MFI) of infected (GFP^+^) versus uninfected (GFP^−^) cells.

### Recombinant Cytokines and Antibodies

Recombinant human MIF (rhMIF) was prepared as described elsewhere (46) (endotoxin content < 0.1 EU/ml). MIF antagonist MIF098 [3-(3-hydroxybenzyl)-5-methylbenzooxazol-2-one] was dissolved in DMSO at a concentration of 149 µM (47). The neutralizing anti-MIF monoclonal antibody (clone NIHlllD.9) was obtained from ascites after purification using protein A/G spin column and resuspended at 5.15 mg/ml (48, 49). A CD74 blocking antibody (BD Pharmingen, clone LN2), the recombinant human cytokines IL-6, IL-8, IL-1β (BioLegend), and TNFα (MiltenyiBiotec), and the cytokine neutralizing antibodies anti-IL-8 (R&D Systems), anti-IL-6, anti-IL-1β, and anti-TNFα (BioLegend) were obtained.

### MDM Stimuli

Monocyte-derived macrophages were infected with the R5-tropic HIV and the infection was left to progress. At day 11, infection percentage was evaluated by p24 intracellular staining as described in the following paragraph (Figure S1 in Supplementary Material). After that, MDMs were washed twice with PBS 1× (Sigma) and rhMIF was added to a final concentration of 1, 10, or 25 ng/ml. Cells were incubated at 37°C for 8 h until the supernatant was collected. When denoted, pretreatment with the αCD74 blocking antibody (or the appropriate isotype control) was performed at 5 ng/ml for 30 min. In some experiments (TLR4 expression), Fc receptors were blocked for 10 min before the addition of the αCD74 blocking antibody (or its isotype-matched control) with an Fc blocking reagent from BD Biosciences.

### Evaluation of TLR4 Expression

After MIF stimulation, infected and uninfected MDMs were harvested and stained with a PE-conjugated anti-TLR4 antibody (BioLegend) for 30 min at 4°C. Following incubation, cells were washed, fixed, and permeabilized using the Cytofix/Cytoperm kit (BD Biosciences) following the instructions provided by the manufacturer. Then, intracellular p24 antigen was stained using an anti-p24-FITC antibody (KC57-FITC, Coulter-clone, Beckman Coulter, USA) for 30 min at 4°C. Cells were then washed, fixed, and acquired in a BD FACSCanto flow cytometer. Data acquisition was performed using the BD FACSDiva software and analyzed subsequently with FlowJO v10 software (Data Analysis Software, LLC). An isotype-matched FITC-conjugated non-specific antibody was used to set the p24-negative population accurately.

First, single cells were gated in a forward scatter (FSC)-height (FSC-H) versus an FSC-area (FSC-A) plot. Then, gating was performed on living MDMs in an FSC versus a side scatter (SSC) plot. Infected cells were identified in an SSC versus FL-1 (FITC) plot as p24 positive events (Figure S1 in Supplementary Material). Bystander cells were identified as the p24-negative population on the same plot. TLR4 median fluorescence intensity (MFI) was determined for uninfected, infected, and bystander cells. Modulation of TLR4 expression was calculated as the ratio between MFI corresponding to infected or bystander versus uninfected cells.

### Cytokine Quantitation

The levels of the following cytokines were evaluated in MDM supernatants using commercially available ELISA sets: IL-8, IL-6, IL-1β, TNFα, IL-10 (ELISA MAX Deluxe kits, BioLegend) and sICAM (DouSet ELISA, R&D Systems). MIF plasma levels were evaluated using an in-house ELISA constructed with an anti-human MIF antibody pair and an MIF standard obtained from BioLegend.

### Permissiveness Induction in Unactivated CD4^+^ T-Cells

Unactivated CD4^+^ T-cells were incubated with supernatants (1/2,000 dilution) from MIF-treated or untreated MDMs, for 72 h at 37°C. Before incubation, supernatants were clarified for 15 min at 600 *g* and UV-inactivated for 30 min (253.7 nm, 15 cm away from the light source). After that, cells were washed and infected with an X4-tropic HIV. Supernatants were collected daily for p24 antigen quantitation till day 7 post-infection. Phytohemagglutinin- (PHA, 2.5 ng/ml, Sigma-Aldrich, USA) and RPMI-treated CD4^+^ T-cells were used as positive and negative controls, respectively.

Alternatively, unactivated CD4^+^ T-cells were stimulated with recombinant IL-1β, IL-6, IL-8, and TNF-α, either alone or in combination, for 72 h prior to infection.

### CD4^+^ T-Cell Phenotype, Viability, and Infection Percentage

The expression of CD38, CD69, HLA-DR, CD25, PD-1, and CD28 surface molecules were analyzed by flow cytometry after CD4^+^ T-cell stimulation with MDMs-derived supernatants for 72 h. Percentages of cells expressing the markers mentioned above as well as their MFI were recorded. Initial gating was performed on lymphocytes followed by gating on CD4^+^ events. Isotype-matched non-specific antibodies were used in each sample to set the corresponding negative populations accurately.

In addition, CD4^+^ T-cells were harvested from day 1 to 7 post-infection and both cell viability and infection percentages were evaluated by flow cytometry. First, single cells were gated in an FSC-H versus an FSH-A plot. Then, living lymphocytes were gated an FSC versus an SSC plot (%viability). Subsequently, infected cells were identified in an FSC-H versus GFP plot (Figure S1 in Supplementary Material). Data acquisition was performed in a BD FACSCanto flow cytometer using the BD FACSDiva software and analyzed subsequently with FlowJO v10 software (Data Analysis Software, LLC).

### Data Analysis

Experiments were performed at least three independent times and analyzed using parametric tests, unless otherwise stated (see exact number of independent experiments in each figure legend). Data normality was assessed using the Shapiro–Wilk test. All tests were considered significant when *p* < 0.05 (GraphPad Prism 7 Software).

## Results

### CD74 Is Upregulated in *In Vitro* HIV-Infected MDMs and This Effect Is Accompanied by Higher MIF Plasma Levels in HIV-Infected Subjects, Compared to HIV-Negative Donors

**Figure 1:**
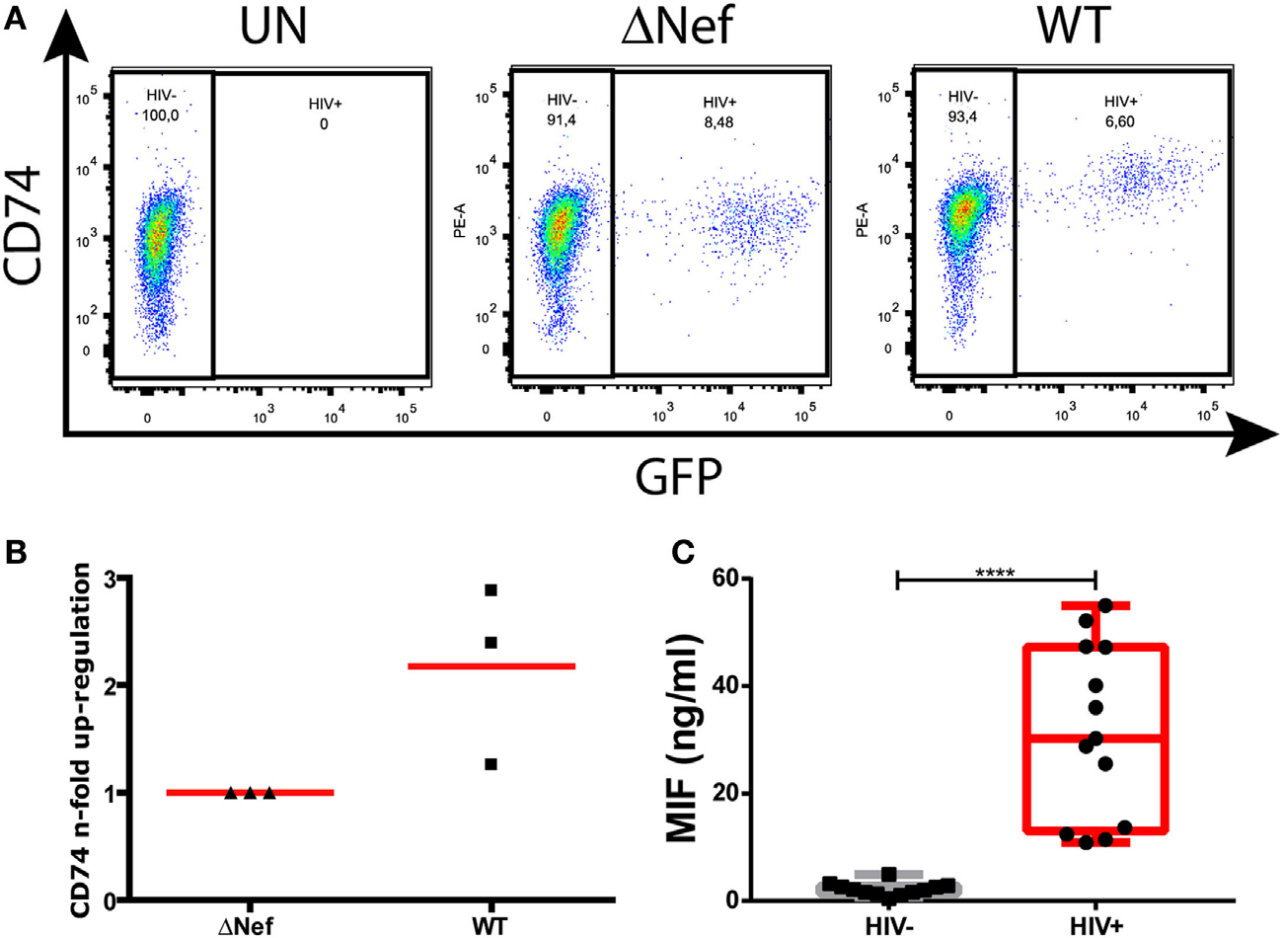
CD74 upregulation in human immunodeficiency virus (HIV)-infected monocyte-derived macrophages (MDMs) and macrophage migration inhibitory factor (MIF) plasma levels in HIV^+^ subjects. **(A)** Flow cytometry analysis of CD74 surface expression in primary uninfected MDMs (left panel); infected MDMs with a Nef-defective virus expressing the reporter molecule GFP (ΔNef HIV-1, middle panel); and infected with a wild type (WT) HIV-1 also expressing the reporter molecule GFP (WT HIV, right panel). The plots show CD74 versus GFP expression (HIV-1 infection) on MDMs [gated previously in a forward scatter (FSC) versus side scatter plot]. In each dot plot, two different populations were gated: the HIV-1 negative population (GFP negative) and the HIV-1 positive population (GFP positive). One representative healthy donor, out of three donors, is shown. **(B)** Quantitation of Nef-mediated upregulation of CD74, calculated as the ratio between FL-2 MFI obtained for cells infected with the WT virus and the FL-2 MFI obtained for cells infected with the ΔNef virus. Each black dot represents one out of three independent experiments (donors). Horizontal red bars stand for the mean value. **(C)** MIF concentration in plasma obtained from HIV-negative (HIV−, *N* = 13) and HIV-positive (HIV+, *N* = 13) donors. Each plasma was evaluated in duplicate. Dots represent the average of duplicates for each donor. Data were normally distributed and analyzed by two-tailed unpaired Student’s *t*-test. Horizontal lines within boxes represent the median and whiskers extend from min to max. ****p < 0.0001.

Nef-mediated CD74 upregulation is a well-described phenomenon. More specifically, this was shown to occur in *in vitro* HIV-infected primary MDMs (13) and in an *ex vivo* model of MDMs obtained from HIV-infected subjects (15). Figure 1A depicts surface CD74 expression in one representative MDM donor. CD74 expression was monitored in uninfected cells (UN, left panel) as well as in cells infected with a Nef-defective virus (ΔNef, middle panel) or a Nef-expressing virus (WT, right panel). In the cultures infected with the WT virus, CD74 MFI was significantly higher in GFP-expressing cells (i.e., infected) when compared to GFP-negative cells (i.e., uninfected). On the contrary, no differences were observed in cultures infected with the Nef-defective virus when comparing infected versus uninfected cells. Figure 1B compiles the upregulation magnitude from three different donors (relative to the Nef-defective virus). On the other hand, it has been reported that the plasma levels of the CD74 ligand MIF were elevated during HIV infection (34, 35). To confirm this, MIF concentration was evaluated in plasma from recently HIV-infected subjects enrolled as part of the *Grupo Argentino de Seroconversión* study group. In line with the previous reports, our results indicated that the MIF plasma level during the acute HIV infection (<6-month post-infection) was 30-fold higher when compared to uninfected individuals (Figure 1C).

### Plasma Membrane Expression of CD74 and CD44, the Signaling Component of the MIF Receptor Complex, Are Increased in WT HIV-Infected MDMs

**Figure 2:**
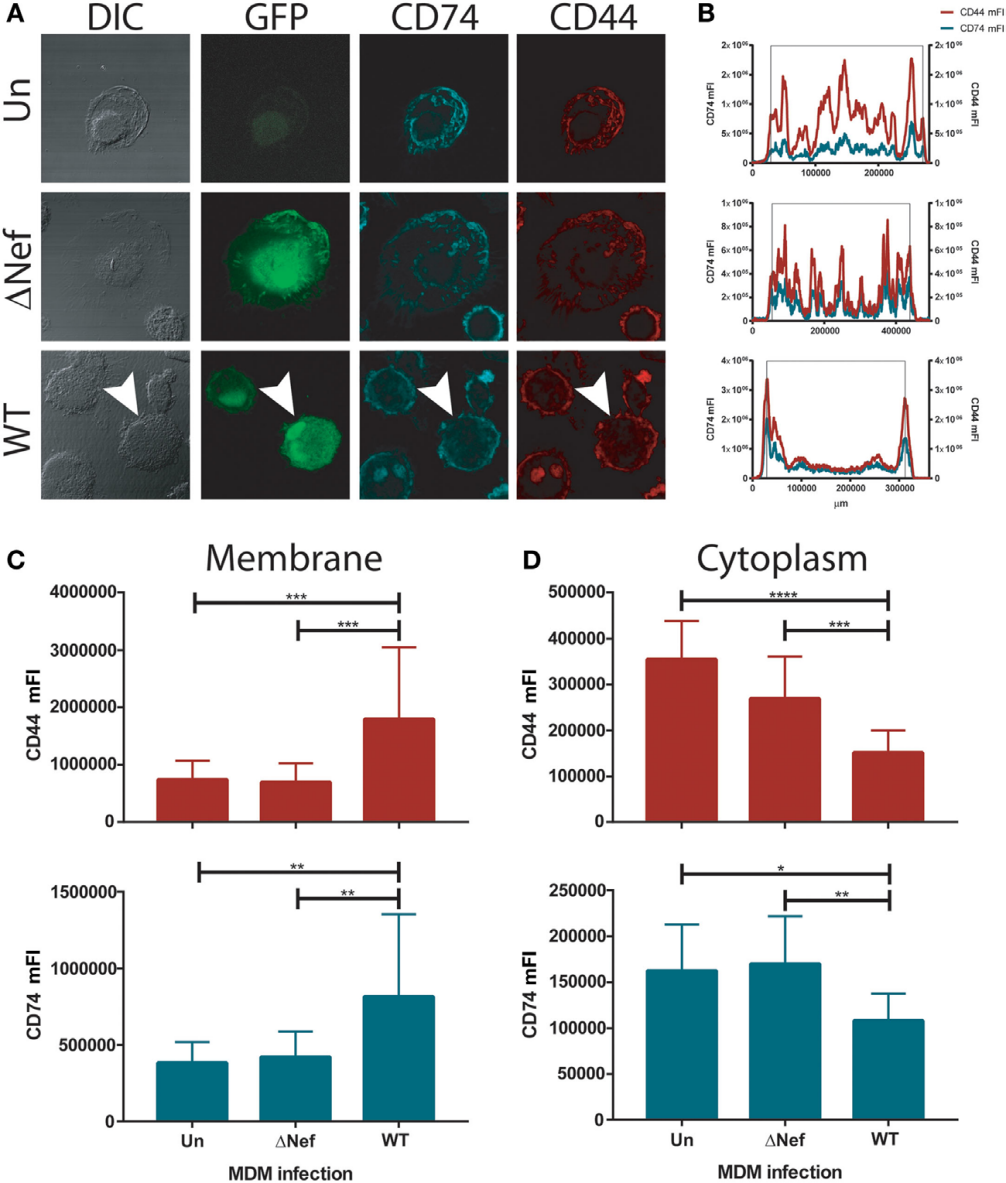
CD74 and CD44 expression in uninfected and infected monocyte-derived macrophages (MDMs). **(A)** Confocal immunofluorescence microscopy of primary uninfected MDMs (UN, upper panels); primary ΔNef human immunodeficiency virus (HIV)-infected MDMs (ΔNef, middle panels); and primary wild type (WT) HIV-infected MDMs (WT, lower panels). From left to right: bright field, GFP (HIV-1 infection), CD74 staining and CD44 staining are shown, in one representative cell for each condition. **(B)** Plots show cross-sectional mean fluorescence intensity (mFI) for CD74 (left axis, cyan line) and CD44 (right axis, red line) corresponding to the depicted cells (in the lower panel, cross-sectional mFI was evaluated in the cell pointed with an arrow). The black lines indicate the area comprising the cell according to the DIC. **(C,D)** Cross-sectional mFI quantification for CD44 (upper panel) and CD74 (lower panel) intensity at plasma membrane **(C)** and cytoplasm **(D)**. Quantifications were performed in 15 individual cells for each condition. Bars represent mean ± SD. Data were analyzed by one-way ANOVA followed by Dunnett’s post-test. *p < 0.05, **p < 0.01, ***p < 0.001, ****p < 0.0001.

We hypothesized that the increased CD74 expression found in HIV-1 infected MDMs may translate into enhanced MIF receptor availability and signal transduction. Thus, the cellular localization of CD74 and the CD44 signaling co-receptor was evaluated in infected primary MDMs by confocal immunofluorescence microscopy. In consonance with previous reports on HeLa-CIITA cells (8, 50), CD74 staining was observed mainly in intracellularly both in uninfected cells and in cells infected with the Nef-defective virus (Figure 2A). More specifically, CD74 staining was mostly located in membranous compartments within the cytoplasm. This could be visualized in the images but it also could be inferred from cross-sectional quantitation of mean fluorescence intensity (mFI) by image processing (Figure 2B) where an uneven mFI profile characterized by different cytoplasmic peaks was obtained. Conversely, an intense CD74 signal comprising the plasma membrane was observed in MDMs infected with the WT virus (Figures 2A,B, lower panels). This is consistent with the ability of Nef to reduce the rate of CD74 internalization (12, 51). Surprisingly, CD44 distribution mirrored that of CD74 in all conditions. Particularly, both CD44 and CD74 were strongly co-expressed at the plasma membrane of WT HIV-infected cells.

In order to quantitate CD44 and CD74 expression at different subcellular localization across all conditions, MFI cross-sectional quantifications corresponding to the regions encompassing only the plasma membrane (Figure 2C) or the cytoplasm (Figure 2D), both for CD74 and CD44, were performed in all infection conditions. Results again indicated that there is a substantial overlap between both molecules in all conditions but that the staining pattern was significantly different in WT-infected cells compared both to uninfected and ΔNef-infected cells, being the expression of both molecules concentrated at the plasma membrane. These data support the notion that the expression of the components of the main MIF receptor might be enhanced in Nef-expressing HIV-infected cells which in turn would be translated into higher responsiveness to MIF by HIV-infected cells.

### MIF Modulates TLR4 Expression in HIV-Infected MDMs in a CD74-Independent Fashion

**Figure 3:**
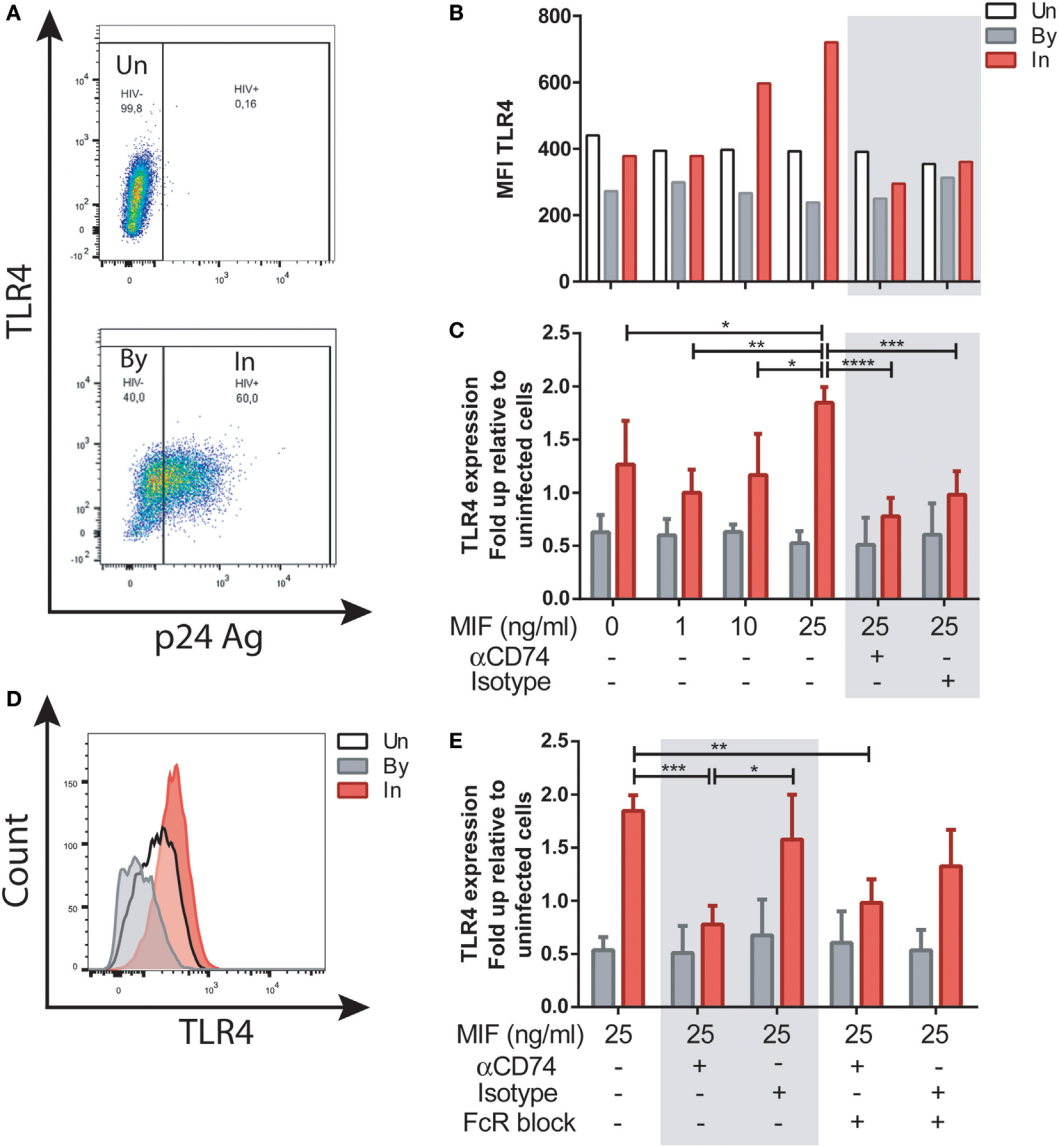
TLR4 expression after macrophage migration inhibitory factor (MIF) stimulation in primary human immunodeficiency virus (HIV)-infected monocyte-derived macrophages (MDMs). **(A)** TLR4 expression in uninfected (UN, upper panel), bystander (By, lower panel), and productively infected cells identified on the bases of intracellular p24 staining (In, lower panel). Living MDMs were gated previously on a forward scatter versus side scatter dot plot. An isotype-matched FITC-conjugated antibody was used to accurately set the p24-negative population. **(B)** TLR4 MFI in uninfected MDMs (Un), in productively infected MDMs (p24 positive population within the well inoculated with the virus, In) and in the bystander uninfected MDMs (p24-negative population within the well inoculated with the virus, By) after MIF treatment. These data represent the results obtained from one representative donor. **(C)** Ratio between the TLR4 MFI of the infected (or bystander population) and the TLR4 MFI of the uninfected cells after treatment with MIF, with or without CD74 blockade with an anti-CD74 antibody. Fold up from four independent donors, evaluated in duplicate are shown collectively. Data represent the mean ± SD. **(D)** Flow cytometry histogram overlay for TLR4 expression on Un, By, and In MDMs all treated with 25 ng/ml MIF **(E)** Ratio between the TLR4 MFI of the infected (or by-stander population) and the TLR4 MFI of the uninfected cells using the different CD74-blocking conditions represented in the *x*-axis. Data were analyzed by two-way ANOVA followed by Tukey’s post-test. *p < 0.05, **p < 0.01, ***p < 0.001, ****p < 0.0001.

We next investigated the well-documented action of MIF to upregulate TLR4 expression in MDMs. To elucidate if this activity was affected by HIV infection, and if it was dependent of CD74 engagement, MDMs were infected with the R5-tropic HIV strain. Uninfected cells were used as controls. At day 11, cells were treated with 1, 10, or 25 ng/ml of MIF. These concentrations were chosen as reported previously to represent those observed in plasma from healthy volunteers, pathophysiological fluids, or plasma from HIV^+^ individuals, respectively (34, 52). In this model, cell viability at day 11 was 83 ± 3.7% in uninfected wells and 66.8 ± 2.5% in infected wells post-treatment. Percentage of infection was 48.48 ± 16.8%, and p24 production in culture supernatant was 20.53 ± 12.7 ng/ml. These parameters did not differed significantly across the different MIF concentrations evaluated here (not shown). To study the expression of TLR4 in uninfected (UN), bystander (By), and productively infected (IN) cells, the gating strategy shown in Figure S1 in Supplementary Material and Figure 3A was used. Representative results from one donor can be observed in Figure 3B. There, it can be observed that TLR4 expression was not modified by MIF treatment either in uninfected of bystander cells. However, TLR4 expression increased with increasing MIF concentration in productively infected cells. When results from four independent donors were expressed relative to their corresponding UN condition and then combined (Figure 3C) it could be observed that TLR4 expression peaked at 25 ng/ml MIF specifically in HIV-infected cells, almost doubling in magnitude when compared to the “no-MIF” condition. This can also be observed in the overlaid histograms shown in Figure 3D, where the MFI for the “IN plus 25 ng/ml MIF” condition is the highest. In order to elucidate whether an interaction between CD74 and MIF was responsible for a higher TLR4 expression, cells were pre-incubated with a neutralizing anti-CD74 antibody (or an isotype-matched control antibody), prior to MIF treatment at peak effect concentration (25 ng/ml MIF). TLR4 expression was significantly reduced both in the CD74-blocked condition but also in the control condition (Figure 3C, gray box). As MDMs may constitutively release MIF in low levels (53), this result may reflect the saturating action of autocrine/paracrine stimulation by endogenously released MIF or, alternatively, a non-specific action of Fc receptor engagement. To test the latter hypothesis, Fc receptors were blocked in this model (Figure 3E). When the FcR block was applied prior to the treatment with the anti-CD74 blocking antibody or the isotype-matched control and the cells were treated with 25 ng/ml MIF, the TLR4 expression reverted to the levels detected in cells only treated with MIF. Moreover, no differences were observed between the CD74 blocked and isotype control conditions. This indicates that the reduction found in TLR4 expression when cells were treated with the anti-CD74 blocking antibody or the isotype-matched control represented a non-specific response to Fc engagement. Collectively, these results indicate that exogenous MIF had an effect on TLR4 up-modulation, which was only evident in IN cells (not UN or By cells) but this effect could not be blocked by interfering MIF binding to CD74.

### Interaction Between CD74 and MIF Triggers the Production of Proinflammatory Mediators Specifically From HIV-Infected MDMs

**Figure 4:**
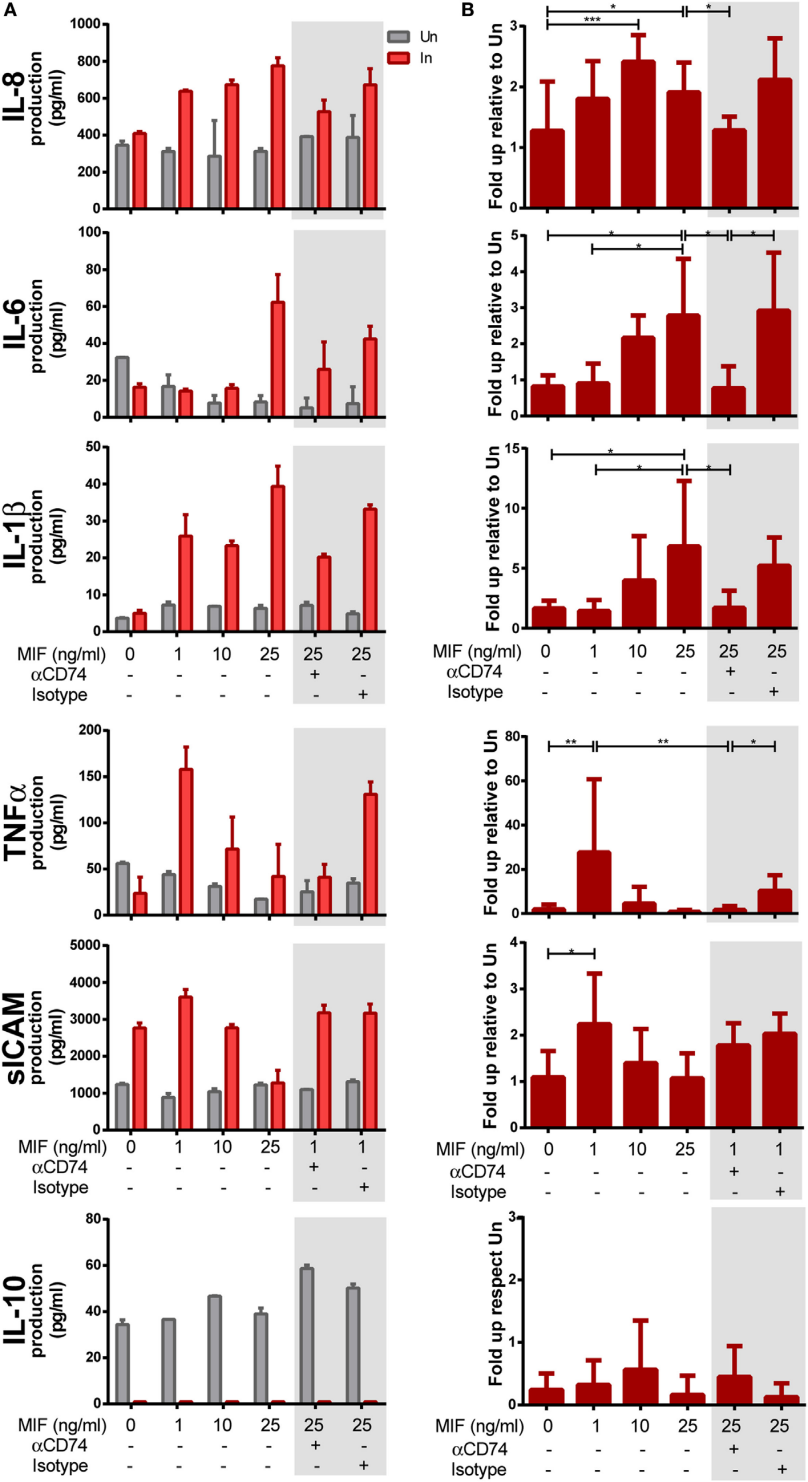
Expression of cytokines after macrophage migration inhibitory factor (MIF) stimulation in primary human immunodeficiency virus (HIV)-infected and uninfected monocyte-derived macrophages (MDMs). **(A)** Expression of IL-8, IL-6, IL-1β, TNF-α, sICAM, and IL-10 in supernatants from HIV-infected (In) and uninfected (Un) MDMs obtained from one representative healthy donor. **(B)** Data combined from six independent experiments (donors), each evaluated in triplicate. Here, data are shown as the ratio between cytokine concentrations found under the infection condition versus the uninfected counterpart. Cells were stimulated with MIF as follows: 0, 1, 10, or 25 ng/ml. Data shown in the gray boxes depict CD74 blocking (10 ng/ml of αCD74 or the corresponding isotype control) followed by MIF stimulation (1 or 25 ng/ml as denoted). Data represent the mean ± SD. Data were analyzed by one-way ANOVA followed by Tukey’s post-test. *p < 0.05, **p < 0.01, ***p < 0.001.

Next, we aimed at investigating the requirement of CD74/MIF interaction in the production of proinflammatory cytokines from HIV-infected versus uninfected MDMs following treatment with MIF. MDMs were infected with the R5-tropic HIV strain and uninfected cells were used as controls. At day 11 (peak infection), cells were treated with different MIF concentrations. Cell viability, infection percentages, and culture supernatant p24 production were as described in the previous section. Figures 4A,B show the raw data from one representative donor and the compiled data from six donors, respectively. Except for IL-10, most supernatants obtained from infected MDMs showed higher production of cytokines when treated with MIF, compared to the uninfected MIF-treated counterpart. While no-MIF effect was observed in uninfected cultures, an MIF-dependent production of IL-8, IL-6, IL-1β, TNF-α, and sICAM was detected in infected cells. Compiled results (*N* = 6) indicated that the greatest effect of MIF on IL-8 production occurred at 10 ng/ml (twofold increase). Similar observations were found for IL-6 (a peak fold increase of 2 and 2.5 at 10 and 25 ng/ml MIF, respectively), IL-1β (a peak fold increase of 8 at 25 ng/ml MIF), TNF-α (a peak fold increase of 26 at 1 ng/ml MIF), and sICAM (a peak fold increase of 2 at 1 ng/ml MIF). By contrast, no-MIF effect was observed in IL-10 production. These results suggest that MIF drive the production of proinflammatory mediators and that this effect is specific for HIV-infected cells. In order to elucidate whether an interaction between CD74 and MIF was responsible for the higher production of these cytokines, cells were pre-incubated with an anti-CD74 blocking antibody prior to MIF treatment at the peak effect concentrations (e.g., 25 ng/ml MIF for IL-8, IL-6, and IL-1β and 1 ng/ml MIF for TNF-α and sICAM; Figures 4A,B, gray boxes). In all cases, except for sICAM, CD74 blockade resulted in diminished levels of cytokine production similar to those observed in the no-MIF condition. Conversely, this effect was not observed when cells were pre-incubated with the corresponding isotype control antibody. Thus, CD74/MIF interaction was a necessary condition for the higher production of the studied cytokines in infected cells. Of note, this result was not recapitulated for sICAM, suggesting an alternative mechanism for this mediator.

Altogether, these results reveal that MIF favors the production of proinflammatory mediators specifically from HIV-infected cells and demonstrate that the interaction with CD74 is needed to achieve this effect. Moreover, they suggest a joint contribution of MDM infection, HIV-mediated upregulation of surface CD74, and MIF stimulation to promote the production of a proinflammatory environment.

**Figure 5:**
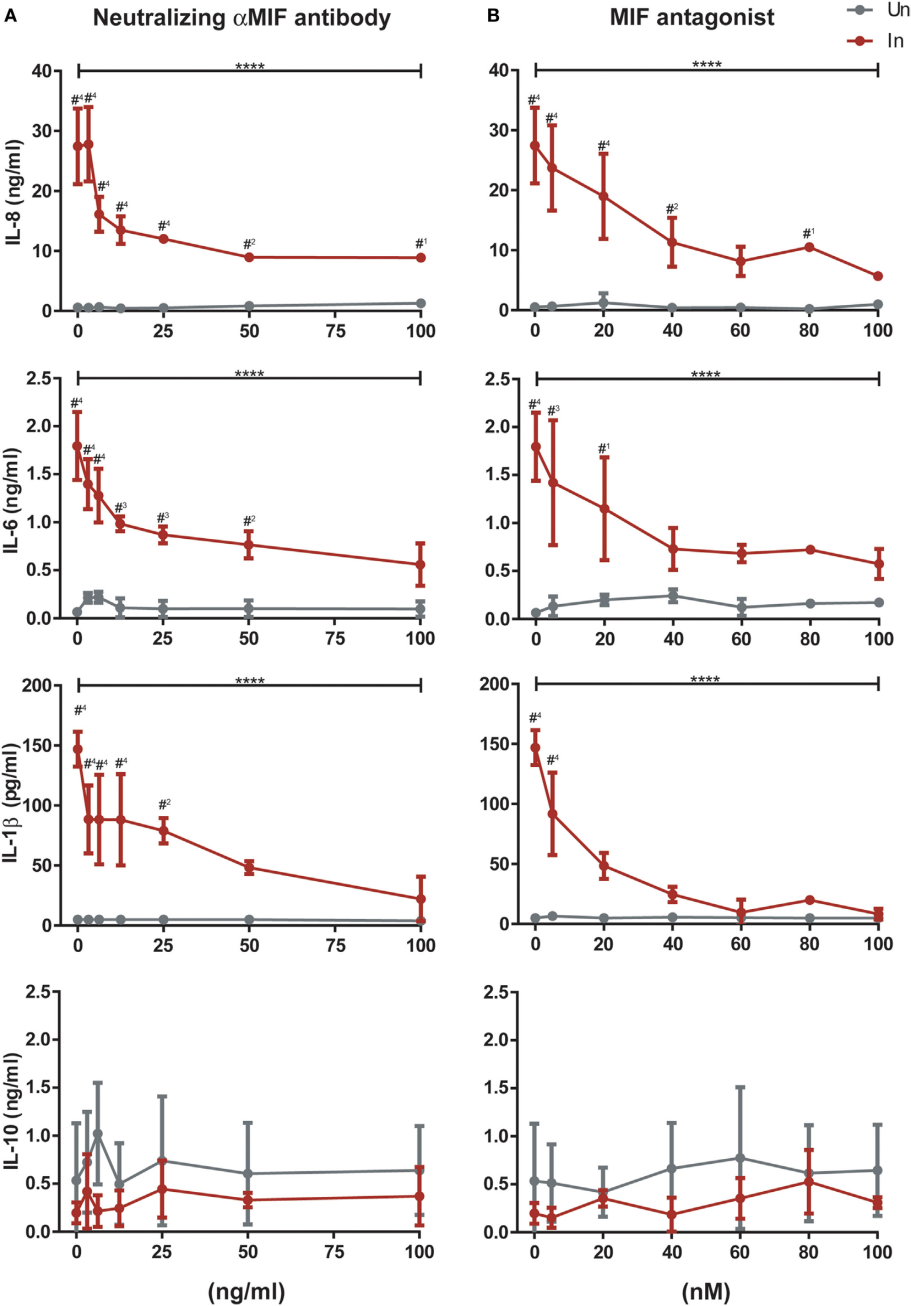
Effect of macrophage migration inhibitory factor (MIF) neutralization in the expression of cytokines from primary human immunodeficiency virus-infected and uninfected monocyte-derived macrophages (MDMs). Serial dilutions of a neutralizing αMIF antibody (clone NIHlllD.9) **(A)**, and the MIF antagonist MIF098 **(B)** were used to inhibit MIF activity in infected (In, red lines) and uninfected (Un, gray lines) MDMs at a constant concentration of this cytokine (25 ng/ml). Data represent three independent experiments (donors), each evaluated in duplicate. Data represent the mean ± SD. Data were analyzed by two-way ANOVA followed by Tukey’s post-test (intragroup analysis, In group only; ****p < 0.0001) or by Sidak’s post-test (intergroup, In versus Un; ^#1^*p* < 0.05, ^#2^*p* < 0.01, ^#3^*p* < 0.001, ^#4^*p* < 0.0001). Asterisks corresponding to the intragroup analysis are shown above the horizontal bars, and those from the intergroup analysis are shown above points corresponding to each antagonist or antibody dilution.

To provide further insight into the role of MIF in these observations, infected and uninfected MDMs from three independent donors were treated with 25 ng/ml of MIF plus different concentrations of an anti-MIF neutralizing monoclonal antibody (range 3.125–100 ng/ml, Figure 5A) or the MIF antagonist MIF098 (range 5–100 nM, Figure 5B). Neither the anti-MIF neutralizing monoclonal antibody nor the MIF antagonist affected cell viability in the concentration range tested. After incubation, production of IL-8, IL-6, IL-1β, and IL-10 was monitored. As expected, a significant reduction in IL-8, IL-6, and IL-1β production was observed upon MIF inhibition when using either the neutralizing antibody or the small molecule MIF antagonist. Serial dilution of both of these CD74/MIF interaction inhibitors reconstituted cytokine expression. Indeed, significant dose-dependent effects were observed, further supporting a role for MIF in promoting the production of proinflammatory mediators specifically from HIV-infected cells. No significant MIF or anti-MIF effects were observed on uninfected cells. Similarly, no changes were observed in IL-10 production.

### CD74/MIF-Dependent Production of Proinflammatory Factors From HIV-Infected MDMs Enhances Viral Production From Unactivated CD4^+^ T-Cells

**Figure 6:**
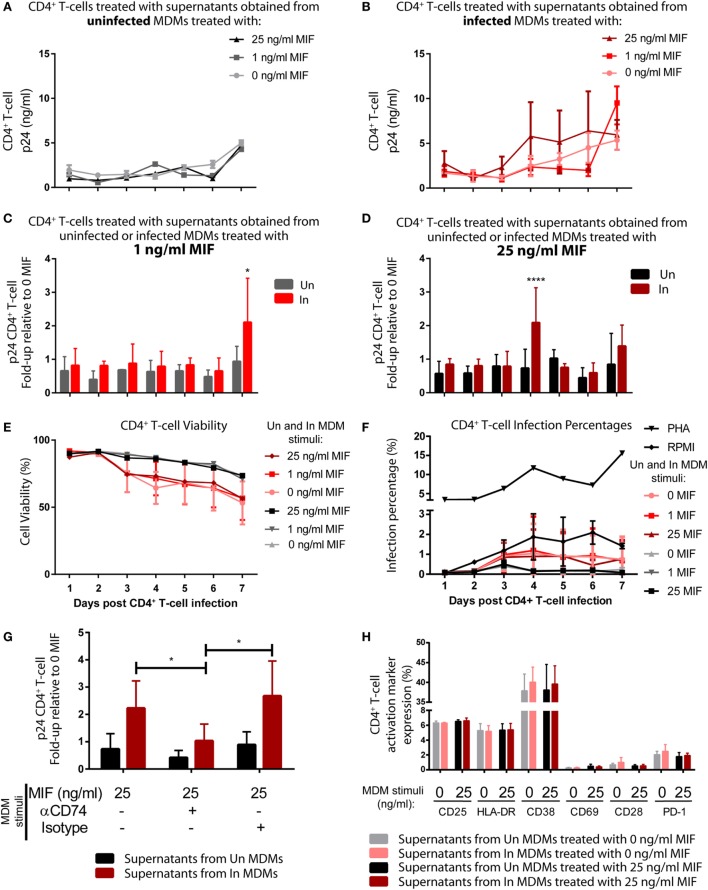
Induction of permissiveness to human immunodeficiency virus type I (HIV-1) infection in primary CD4^+^ T-cells after stimulation with macrophage migration inhibitory factor (MIF)-treated monocyte-derived macrophages (MDMs)-derived supernatants. **(A,B)** Seven-day kinetics of HIV p24 antigen production from primary unactivated CD4^+^ T-cell incubated with supernatants from uninfected **(A)** and infected **(B)** MDMs treated with 0, 1, or 25 ng/ml MIF. **(C,D)** Ratio of p24 production from unactivated CD4^+^ T-cell incubated with supernatants from uninfected MDMs and infected MDMs treated with 1 ng/ml MIF **(C)** or 25 ng/ml MIF **(D)** over the no-MIF condition. **(E)** Percentage of living CD4^+^ T-cells stimulated with supernatants derived from uninfected (black, dark gray, and light gray lines) and infected (pink, red, and dark red lines) MDMs. **(F)** Percentage of infected (GFP^+^) CD4^+^ T-cells after stimulation with MDM-derived supernatants obtained from MIF-treated uninfected MDMs (black, dark gray, and light gray lines), infected (pink, red, and dark red lines) MDMs, RPMI (negative control, black line with diamonds), or PHA (positive control, black line with triangles). **(G)** Ratio of p24 production from unactivated CD4^+^ T-cell incubated with supernatants from uninfected MDMs and infected MDMs treated with 25 ng/ml MIF, with or without CD74 blockade with an anti-CD74 antibody. **(H)** Expression of surface markers on CD4^+^ T-cells subjected to 72 h stimulation with supernatants derived from infected and uninfected MDMs and exposed or not to MIF treatment (0 and 25 ng/ml MIF). Data represent the mean ± SD from six independent donors evaluated in duplicate. In **(C,D)**, data were analyzed by two-way ANOVA followed by Sidak’s post-test. In **(G)**, data were analyzed by two-way ANOVA followed by Tukey’s post-test. *p < 0.05, ****p < 0.0001.

Then, we reasoned that conditioned media (supernatants) from MIF-treated HIV-infected MDMs could have an enhancing effect on the permissiveness of unactivated CD4^+^ T-cells to HIV infection, which are mostly naturally resistant to HIV. To test this hypothesis, supernatants obtained from infected and uninfected MDMs, treated or not with MIF, were UV inactivated and used to stimulate purified unactivated CD4^+^ T-cells. RPMI- or PHA-treated CD4^+^ T-cells were used as negative and positive controls, respectively. At 72 h post-treatment, CD4^+^ T-cells were infected with an X4-tropic GFP-expressing viral strain, and infection was monitored during 7 days by flow cytometry to evaluate % of infected cells (GFP+, see Figure S1 in Supplementary Material) and by ELISA (p24 antigen) to evaluate viral production. Figures 6A,B depict the viral production kinetics observed in CD4^+^ T-cells treated with supernatants derived from uninfected and HIV-infected MDMs, respectively, treated with 0, 1, and 25 ng/ml MIF (the 10 ng/ml condition was not evaluated). Viral production was very low when CD4^+^ T-cells were pre-incubated with supernatants derived from uninfected MDMs and it was independent of MDM MIF treatment (Figure 6A). A similar kinetic was observed in CD4^+^ T-cells pre-incubated with supernatants from infected MDMs treated with 0 MIF (Figure 6B, pink line). Conversely, viral production from CD4^+^ T-cells pre-incubated with supernatants from infected MDMs treated with 1 ng/ml MIF (Figure 6B, red line) and 25 ng/ml MIF (Figure 6B, dark red line) tended to increase over time reaching maximal viral production at day 7 for the 1 ng/ml MIF condition and at day 4 for the 25 ng/ml MIF condition. This can be better observed in Figures 6C,D. Here, viral production from CD4^+^ T-cells pre-incubated with supernatants from infected MDMs treated with 1 ng/ml (Figure 6C) and 25 ng/ml (Figure 6D) is shown relative to the viral production from CD4^+^ T-cells pre-incubated with supernatants from uninfected MDMs treated with the corresponding MIF concentration. Overall, results indicate that, at 7 days post-infection, viral production from initially unactivated CD4^+^ T-cells is significantly higher upon exposure to supernatants derived from infected MDMs treated with 1 ng/ml MIF, compared to exposure to supernatants derived from uninfected MDMs. Similarly, unactivated CD4^+^ T-cells sensitized with supernatants from infected MDMs treated with 25 ng/ml MIF showed a significant, albeit transient, increase in viral production at day 4 post-infection, compared to the uninfected condition, which was later downmodulated.

Despite sustained viral production is observed from CD4^+^ T-cells treated with supernatants derived from the 25 ng/ml MIF-treated infected MDMs (see Figure 6B, dark red line), the peak effect observed at day 4 in Figure 6D is lost at later time points. This might be indicating that the supernatant from infected MDMs treated with 25 ng/ml might be enhancing cell permissiveness and/or favoring an earlier viral production from these unactivated cells.

In order to rule out the possibility of inefficient viral inactivation by UV of MDM-derived supernatants, CD4^+^ T-cells were incubated with the UV-inactivated supernatants and left to proceed as described previously but without infecting them. At days 4 and 7, p24 antigen was quantified, and no viral production was detected under either condition. This indicates that no viral carry-on from infected MDMs occurred. We also explored whether CD4^+^ T-cell viability and infection percentages were affected by the addition of supernatants derived from MDMs treated under the different MIF conditions. No differences in cell viability (Figure 6E) was observed along time for CD4^+^ T-cell treated with supernatants obtained from uninfected MDMs treated with the different MIF concentrations (black, dark gray, and light gray lines). As expected, CD4^+^ T-cell treated with supernatants obtained from infected MDMs showed a reduction in cell viability, compared to the uninfected condition, no differences were observed across the different MIF concentrations (pink, red, and dark red lines). This indicates that cell viability most likely does not account for the differences observed in viral production. On the other hand, the infection percentage was higher after PHA treatment (Figure 6F). All other conditions, including the RPMI control, showed infection percentages <2% and no significant differences across treatments were observed. This might be indicating that the treatments with the MDM-derived supernatant might enhance viral production from those few infected cells rather than promoting infection. Then, the effect of blocking CD74/MIF interaction in MDMs on the observed results was studied. For this, unactivated CD4^+^ T-cells were incubated with supernatants derived from MDMs in which the interaction between CD74 and MIF had been blocked with an anti-CD74 neutralizing antibody (Figure 6G). This assay was performed using supernatants from MDMs treated with the 25 ng/ml condition and viral production was evaluated at day 4 to reproduce the peak result observed in Figure 6D. Notably, unactivated CD4^+^ T-cells treated with supernatants derived from “CD74-blocked” MDMs were not able to recapitulate the increase in viral production observed in the “non-blocked” condition. In summary, these results suggested that soluble factors released after MIF treatment in infected MDMs enhanced permissiveness of unactivated CD4^+^ T cell. Moreover, the production of these factors appears to be dependent on CD74/MIF interaction as the effect was abrogated by immunoneutralization of CD74.

Finally, we investigated if preincubation with the supernatants derived from MDMs induced CD4^+^ T-cell activation differentially as it is known that the level of cell activation correlates with HIV-1 infection efficiency. To assess this, the phenotype of CD4^+^ T-cells was studied after stimulation with MDMs-derived supernatants. The expression of CD38, CD69, HLA-DR, CD25, PD-1, and CD28 markers was evaluated by flow cytometry after 72 h. Supernatants from infected or uninfected MDMs treated (25 ng/ml) or not with MIF were used. No differences were detected in the percentage of cells expressing the different membrane markers (Figure 6H) or their MFI (not shown) among treatments. In sum, the improved viral production observed in CD4^+^ T-cells after treatment with supernatants derived from 25 ng/ml MIF-treated infected MDMs could not to be explained by differential cell viability, infection percentage, or cell activation (measured by surface markers).

**Figure 7:**
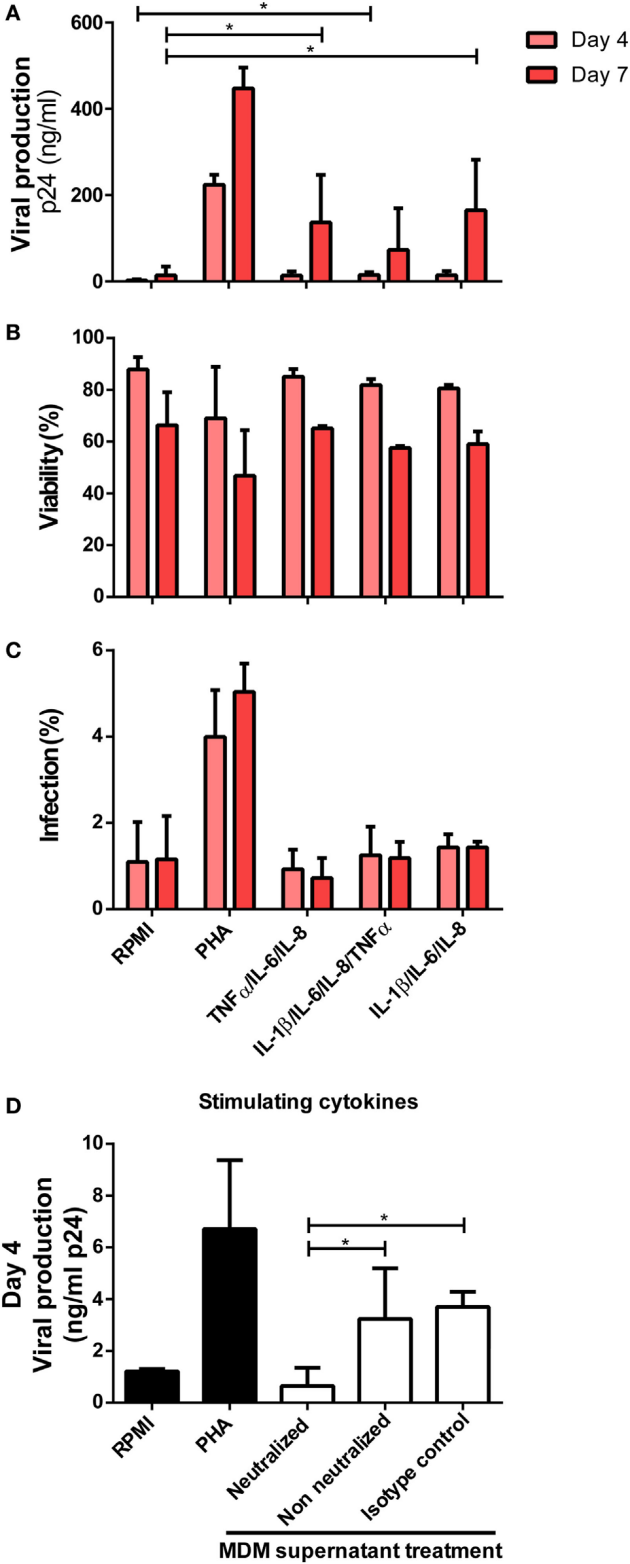
Identification of cytokines as responsible for enhancing human immunodeficiency virus type I (HIV-1) infection in unactivated CD4^+^ T-cells. **(A)** Unactivated CD4^+^ T-cells were stimulated with different combinations of cytokines for 72 h. Then, cells were infected and p24 antigen production was evaluated at days 4 and 7 post-infection. Each condition was compared with the corresponding RPMI condition (negative control). As a positive control, PHA stimulation was used. Percentage of living CD4^+^ T-cells **(B)** and percentage of infected (GFP^+^) CD4^+^ T-cells **(C)** after stimulation with the denoted treatments are shown. Data represent mean ± SD from four independent donors evaluated in duplicate. Concentrations of cytokines used in these experiments corresponded to the average concentrations found in monocyte-derived macrophage (MDM) supernatants stimulated with 25 ng/ml macrophage migration inhibitory factor (MIF) (peak effect) as follows: 250 pg/ml IL-6, 9,000 pg/ml IL-8, 1,400 pg/ml TNF-α, and 20 pg/ml IL-1β. **(D)** Neutralization of IL-8, IL-6, IL-1 β, and TNFα biological activity with monoclonal neutralizing antibodies. Primary CD4^+^ T-cells were incubated with supernatants derived from the 25 ng/ml MIF-treated HIV-infected MDM neutralized previously with 18 µg/ml anti-IL-8, 20 ng/ml anti-IL-6, 2 µg/ml anti-IL-1β, and 2 µg/ml anti-TNFα antibodies. Non-neutralized and isotype control antibody conditions were tested for comparison. Also, RPMI and PHA controls were included. Viral production was evaluated at day 4 post-infection. Data were analyzed by one-way ANOVA followed by Dunnett’s post-test (all conditions versus the corresponding RMPI control) in **(A)** and Tukey’s post-test in **(D)**. *p < 0.05.

In summary, we identified that the production of TNFα, IL-6, IL-8, and IL-1β increased significantly after CD74/MIF interaction in infected MDMs. Moreover, conditioned media from MIF-treated infected MDMs significantly enhanced viral production from unactivated CD4^+^ T-cells. Thus, the next step was to study a possible link between cytokines secreted by infected MDMs in an MIF-dependent manner and viral production from unactivated CD4^+^ T-cells. To do this analysis, recombinant IL-1β, IL-6, IL-8, and TNFα were used to stimulate primary unactivated CD4^+^ T-cells in the absence of any other stimuli at concentrations that resemble those found in MDM supernatants stimulated with 25 ng/ml MIF (peak effect). RPMI alone and PHA-supplemented RPMI were used as negative and positive controls, respectively. After 72 h, cells were infected, and viral p24 antigen was quantified. CD4^+^ T-cells treated with single cytokines or dual combinations did not alter viral production, regardless of the cytokines involved (data not shown). Only when treating CD4^+^ T-cells with three or four cytokines simultaneously viral production increased significantly compared to the RPMI control (Figure 7A). No differences in cell viability and infection percentages were observed across treatments (except for PHA) (Figures 7B,C). Finally, MDMs-derived supernatants were incubated with anti-IL-8, -IL-6, -IL-1β, and -TNFα neutralizing antibodies and used as CD4^+^ T-cells activation stimuli. In line with our hypothesis, a significant reduction in viral production was observed under this condition, compared to the non-neutralized and isotype control supplemented supernatants (Figure 7D).

Overall, IL-1β, IL-6, IL-8, and TNFα were identified as factors secreted from MIF-treated HIV-infected MDMs that, in combination, exerted a transient enhancing effect on viral production from unactivated CD4^+^ T-cells.

### *In Vitro* Infections With Transmitted/Founder (T/F) HIV Strains Reproduced Both the Effect of MIF on the Production of Proinflammatory Mediators From HIV-Infected MDMs and Also the Enhanced Viral Production From Unactivated CD4^+^ T-Cells Stimulated With Conditioned Media Derived From MIF-Treated HIV-Infected MDMs

To examine whether the findings reported here could be extended to other HIV-1 strains, we generated viral stocks from selected transmitted/founder (T/F) IMCs. These clones were derived from full-length transmitted HIV-1 genomes and represent viruses actually responsible for productive clinical infection. Thus, these are instrumental tools for studying different aspects of HIV pathogenesis (36–39).

**Figure 8:**
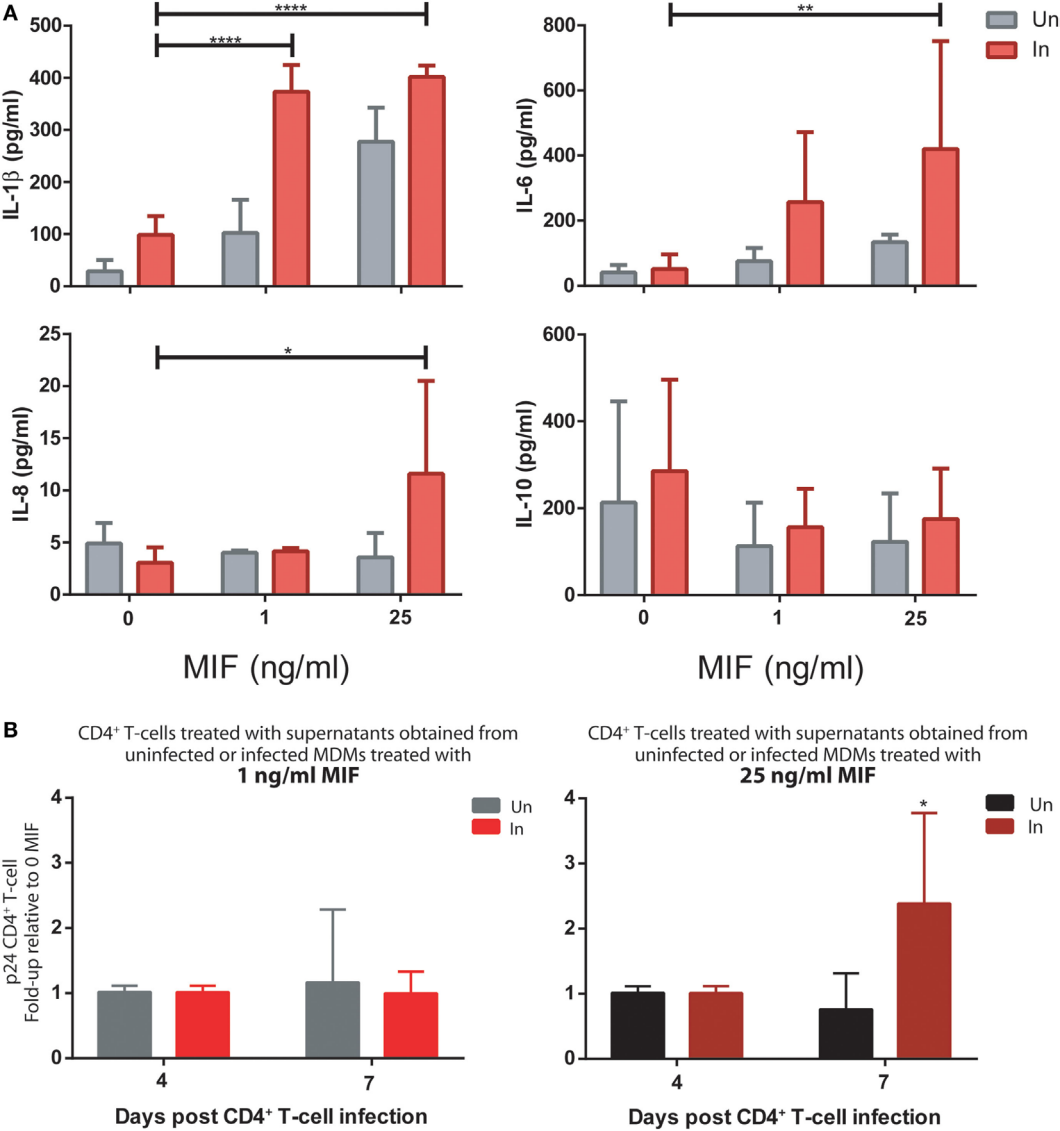
Infection of monocyte-derived macrophages (MDMs) and unactivated CD4^+^ T-cells with T/F viruses reproduce the results obtained with the R5-tropic (BAL) and the X4-tropic (NL4-3) laboratory strains. **(A)** Expression of IL-1β, IL-8, IL-6, and IL-10 in supernatants from uninfected (Un) and R5-tropic T/F-infected (In) MDMs. Data represent mean ± SD from three independent donors. Data were analyzed by two-way ANOVA followed by Tukey’s post-test. *p < 0.05, **p < 0.01, ****p < 0.0001. **(B)** Human immunodeficiency virus (HIV) p24 antigen production from primary unactivated CD4^+^ T-cells incubated with supernatants from uninfected and infected MDMs treated with 1 ng/ml (left panel) or 25 ng/ml (right panel) macrophage migration inhibitory factor (MIF). Treated CD4^+^ T-cells were infected with a dual-tropic T/F virus and viral production was evaluated at 4 and 7 days post-infection. Data represent mean ± SD from five independent donors. Data were analyzed by two-way ANOVA followed by Sidak’s post-test. *p < 0.05.

First, MDMs were infected with the R5 T/F virus. At day 11, both infected and uninfected cells were treated with 0, 1, or 25 ng/ml MIF and the production of cytokines was evaluated in cell supernatants. As observed for the HIV BAL strain, an MIF-dependent effect was observed for IL-1β, IL-6, and IL-8, which was significantly marked in infected cells while the production of IL-10 was unaltered across conditions (Figure 8A). In particular, peak IL-6 and IL-8 effects were observed at 25 ng/ml MIF while for IL-1β, the effect was already evident at 1 ng/ml MIF. Contrary to our initial observations using the BAL strain, the production of TNF-α and sICAM was not affected by MIF (not shown).

Then, the effect of MDM supernatants on viral production from unactivated CD4^+^ T-cells was again tested but using the dual-tropic T/F virus to infect the CD4^+^ T-cells. Thus, supernatants from MIF-treated BAL-infected and uninfected MDMs were used to stimulate unactivated CD4^+^ T-cells during 72 h. Viral production was evaluated at 4 and 7 days post-infection. Results indicated that production of a dual-tropic T/F virus from unactivated CD4^+^ T-cells sensitized with supernatants derived from 25 ng/ml MIF-treated infected MDMs was significantly higher compared to the uninfected counterpart (Figure 8B, right panel). Contrary, no effect was observed when using supernatants derived from 1 ng/ml MIF-treated MDMs (Figure 8B, left panel). These results partially recapitulated those obtained when infecting unactivated CD4^+^ T-cells with the X4-tropic NL4-3 laboratory strain: an enhancing effect on viral production was observed when sensitizing cells with MIF-treated infected MDM-derived supernatants although the kinetics seems to be different for the T/F strain.

Overall, MIF effect on the production of proinflammatory mediators from HIV-infected MDMs and also the enhancing effect of the conditioned media (derived from MIF-treated HIV-infected MDMs) on viral production from unactivated CD4^+^ T-cells could be reproduced when using T/F viral strains. This provides further support to the notions presented in this work pointing toward a relevant role of the MIF/CD74 axis in HIV pathogenesis.

## Discussion

It has become increasingly clear that signaling events downstream of MIF/CD74 interaction are key components in the regulation of immune responses that are involved in the pathogenesis of different inflammatory and immune-mediated diseases. However, whether this axis participates in HIV-mediated immune dysfunction has not been elucidated yet. Several lines of evidence suggest that this might be the case, based on the fact that CD74 expression is modulated in HIV-infected cells and that MIF plasma levels are elevated throughout the course of infection in HIV-infected subjects. Results depicted in this study provide support to this hypothesis by showing that production of soluble inflammatory factors by primary HIV-infected MDMs was increased in an MIF dose-dependent manner and that CD74/MIF interaction was necessary for this effect. Moreover, the conditioned environment generated by MIF/CD74 interaction in infected MDMs promotes CD4^+^ T-cell permissiveness to infection.

In an initial report, CD74 was described as the central component of the MIF cell surface receptor (54). However, whereas the CD74 intracellular domain was shown to undergo intracellular phosphorylation upon engagement of the CD74 ectodomain by MIF, its short non-canonical structure suggested the involvement of a recruited co-receptor. A subsequent study demonstrated that CD44 was a necessary component for MIF signaling (16). CD74 surface expression has been shown to be upregulated in HIV-infected cells, and we show herein that this was also accompanied by surface CD44 upregulation which translated into an overlapping cell surface expression pattern observed specifically in WT HIV-infected cells. This allowed us to hypothesize that this phenomenon may translate into higher MIF receptor availability and enhanced receptor activation by MIF in these cells. It is worth highlighting that only few reports describe the effect of HIV infection in CD44 expression in myeloid cells (55–57). Other molecules proposed to act as MIF coreceptors together with CD74, including CXCR2, CXCR4, and CXCR7 (17, 18, 58), were not analyzed here.

This evidence led us to study the MIF/CD74 interaction in HIV infection. First, we decided to study MIF-mediated modulation of TLR4 in infected cells. This was based on the observation that detectable plasma LPS levels are common in HIV infection (2, 59), thus modulation of one of the components of LPS receptor complex, TLR4, might contribute to disease progression. Also, endogenous MIF has been shown to modulate TLR4 expression in murine macrophages (60, 61). Here, the effect of exogenously added MIF was studied to unravel how its interaction with CD74 might have an impact on TLR4 modulation. In our system, a modest effect was observed particularly in infected cells at 25 ng/ml MIF with no evidence of CD74 participation. A more recent report indicated that exogenously added MIF could modulate TLR4 in murine fibroblasts but only at 375 ng/ml MIF (15-fold higher concentration than in our system) (62). Regardless MIF stimuli, it is also worth pointing that TLR4 expression was lower in bystander cells, compared to the uninfected condition. We speculate that this might be the consequence of factors produced by productively infected cells that affect, directly or indirectly, the phenotype and/or function of the neighboring non-productively infected cells (63).

The capability of HIV-infected MDMs to secrete different proinflammatory cytokines in response to MIF treatment was examined later. The fact that MIF is able to stimulate the secretion of proinflammatory cytokines in different settings is a phenomenon well-documented (19, 64–66). Moreover, many of these events have been reported to occur after CD74 engagement and by activating multiple intracellular signaling pathways (7, 29, 67–69). We add new evidence on the role of MIF in the clinically important HIV infection scenario. Quantitation of IL-1β, IL-6, IL-8, TNFα, and sICAM in MDMs supernatants demonstrated that MIF stimulation led to an augmented production of the proinflammatory cytokines studied. The first three cases showed a dose dependence with the MIF stimuli with maximum expression when using the highest MIF concentration tested. On the other hand, TNFα and sICAM production peaked at the lowest MIF concentration tested. Even more, enhanced production of IL-1β, IL-6, and IL-8 was also observed in MIF-treated MDMs infected with a T/F virus, indicating that this effect could be reproduced with clinically relevant viral strains. These results led to a direct link between the secretion of proinflammatory cytokines and MDM exposure to MIF. Even more relevant, the effect was maximum in infected cells compared to uninfected cells, pointing to a differential effect on HIV-infected cells. According to our hypothesis, this outcome could be explained by the higher availability of membrane CD74 molecules in HIV-infected cells that translate into greater MIF binding and signal transduction. To confirm that the MIF/CD74 axis was required for these effects, the interaction was blocked with a αCD74 immunoglobulin. The production of most mediators (all but sICAM) was inhibited by this treatment. In sum, our results provide support to the hypothesis that links the MIF/CD74 interaction and the differential production of proinflammatory molecules such as IL-1β, IL-6, IL-8, and TNFα expression from HIV-infected cells. Of note, sICAM was proposed to promote interactions between B and T cells that ultimately render resting T-cells permissive to HIV infection (70). Thus, the finding regarding MIF-mediated induction of sICAM production was of special interest. Results indicated that sICAM response peaked at 1 ng/ml MIF and was then downmodulated at higher exogenous MIF concentrations. Again, this result might be reflecting the saturating action of autocrine/paracrine stimulation by endogenously produced MIF or the involvement of alternative mechanisms yet to be elucidated.

During the last years, the concept of macrophage polarization has gained special focus, thus distinguishing different MDM subsets with different functionalities (71). Here, unpolarized (i.e., differentiated from blood monocytes only in the presence of GM-CSF) MDMs were used throughout the study. This was based on bibliography indicating that M1 and M2 polarized MDMs are less efficient to support productive HIV infection compared to unpolarized cells due to different blocks imposed at different levels of the replicative cycle (72–74). On the other hand, it has been reported that MDM infection with HIV results in polarization toward an M1-like phenotype. Moreover, infection sensitized macrophage responses to TLR ligands (75). Despite TLR ligands were not assayed here, a parallelism between these and our findings can be proposed since, according to our results, HIV infection renders MDMs more reactive to a proinflammatory stimuli such as MIF.

The hallmarks of HIV infection include the gradual decline in the number of CD4^+^ T-lymphocytes and the chronic and persistent inflammation and immune activation. HIV-mediated immunopathogenesis is a complex process involving a dynamic interplay between viral and host molecules. Activation of T cells is driven directly by HIV replication but also by indirect mechanisms such as the breakdown in the gut mucosa and dysfunction of immunoregulatory factors, among others (2). Concomitantly, immune activation plays a key role in the systemic spread of the infection. HIV efficiently infects activated CD4^+^ T-cells leading to a productive infection state. However, it has been recently documented that the infection of unactivated CD4^+^ T-cells also occurs, resulting mostly in a latent infection (76). We therefore raised the question of whether MIF-treated MDM-derived supernatants could promote the infection of unactivated primary CD4^+^ T-cells in the absence of other stimuli. Results indicated that viral production was significantly enhanced by conditioned media obtained from MIF-treated HIV-infected MDMs (compared to MIF-treated uninfected MDMs). The effect was highest as early as 4 days post-infection when using the 25 ng/ml MIF-stimulated MDMs while it occurred at day 7 post-infection for the 1 ng/ml MIF condition. This pattern in viral production mirrors the MIF-dependent cytokine production from infected MDMs: a peak production of IL-1β, IL-6, and IL-8 was observed with 25 ng/ml MIF and a peak in TNFα with 1 ng/ml. Particularly, TNFα showed the highest modulation magnitude when infected MDMs were compared with those uninfected but at the lowest MIF concentration tested. In line with the dependence on CD74/MIF interaction for MDM cytokine production, blockade of MIF/CD74 engagement in infected MDMs abrogated the effect observed in CD4^+^ T cells. In order to better support the impact of cytokines produced from infected MDMs downstream of the MIF/CD74 interaction on the permissiveness of unactivated CD4^+^ T-cells, recombinant cytokines were used as direct stimuli. When a combined treatment with IL-1β, IL-6, IL-8, and/or TNFα was attempted, viral production increased. In the same line, neutralizing the biological activity of these cytokines in MDM-derived supernatants resulted in diminished CD4^+^ T-cell permissiveness, resembling the same scenario obtained in the negative control. Finally, supernatants from MIF-treated HIV-infected MDMs could enhance viral production from unactivated CD4^+^ T-cells infected with a T/F virus suggesting that the proposed mechanism extends not only to laboratory strains but also to primary viral isolates.

The fact that cytokines enhance viral replication but, more importantly, promote the infection of resting CD4^+^ T-cells is not new (77). CCL19, CCL21, IL-7, and IL-15 are known to promote latent infection in resting CD4^+^ T-cells (78–80). Also, IL-6 and TNFα has been shown to facilitate infection of resting CD4^+^ T-cells and to induce productive infection (77, 81). In a particularly relevant report, soluble factors (sCD23 and sICAM) released by infected MDMs promoted the efficient infection of resting lymphocytes although the presence of B cells was a requisite for this effect (70). Nevertheless, it resulted interesting that the effect on resting CD4^+^ T-cell permissiveness mediated by sCD23 and sICAM, occurred without promoting cell activation and proliferation, which is in line with the observations described in this work. In a recent report, Morris et al. (81) described that IL-6 produced from endothelial cells increased productive HIV infection in resting CD4^+^ T-cells. Even more, this effect was not accompanied by an increase in the expression of T cell activation markers, mirroring our own results. Although the mechanism underlying this phenomenon was not studied by the authors, it could be associated with the capacity of IL-6 to favor CD4^+^ T-cell cycling and survival (82). On the other hand, IL-8 and TNFα have been reported to directly enhance the rate of productive infection in activated T cells (82, 83). In particular, binding of TNFα to its receptor triggers several signaling cascades, including NF-κB, MAPK, ERK, and JNK pathways, which directly enhances transcription from the LTR promoter both on models of productive HIV infection and also in latently infected cells [reviewed in Ref. (82)].

Thus, in our model we suggest that IL-6, IL-8, TNFα, and IL-1β might be acting synergistically at different levels (i.e., modifying the cellular environment and/or by enhancing transcriptional and/or post-transcriptional mechanisms) to promote, at least transiently, the productive infection of unactivated CD4^+^ T cells. This could explain that no effect was observed when cells were treated with a single or a dual combination of the cytokines studied. It will be a matter of subsequent studies to investigate if this also results in a higher rate of latent infection in these cells. Finally, the role of IL-1β is less clear since there is no definite report suggesting a direct mechanism of IL-1β-mediated modulation of HIV replication in T-cells.

Taken together, we postulate that modulation of CD74 by HIV infection in MDMs leads to the enhanced susceptibility of these cells to MIF stimulation, which may have an impact on the spread of HIV infection and the enhancement of viral-mediated pathogenesis. The current results indicate that the expression of proinflammatory cytokines was significantly higher in MIF-stimulated infected MDMs compared to MIF-stimulated uninfected MDMs. In addition, this proinflammatory microenvironment, conditioned positively primary unactivated CD4^+^ T cells to HIV-1 infection. The methodological strengths of this work include the exclusive use of primary cells, emphasizing that treatment effects can be observed despite interdonor variability, the use of both laboratory and T/F viral strains, and the employment of physiological MIF concentrations as stimuli. On the other hand, interdonor variability and the use of a limited number of donors might have masked differences across conditions, representing an important limitation of the study. Also, macrophages exhibit significant heterogeneity *in vivo*, as already discussed and this fact should not be overlooked. Thus, results might not be extended to polarized MDMs, tissue macrophages, or other HIV susceptible myeloid cells such as dendritic cells. For instance, it would be very interesting to evaluate CD74/MIF axis in microglial cells. If a similar hypothesis was confirmed in this model, this mechanism could be associated with the development of HIV-related neurological complications.

Overall, this work provides further insights in the role of macrophages in HIV infection, not only as a cell type which supports viral replication itself but also as a source of soluble factors that facilitate viral dissemination. Evidence gathered here suggests that CD74/MIF interaction could be implicated in modulating viral reservoir seeding, persistent viremia and inflammation—all key aspects of HIV immunophatogenesis. Data presented here support further studies to fully understand how this mechanism operates in HIV infection and to explore the possibility to target CD74/MIF axis as a therapy aimed at reducing inflammation and reservoir size during HIV infection.

## Ethics Statement

Samples from HIV-infected subjects used in this study were enrolled as part of an ongoing acute/early primary HIV infection cohort from Argentina (Grupo Argentino de Seroconversión study group). This study was reviewed and approved by two institutional review boards (IRB): Comité de Ética Humana, Facultad de Medicina, Universidad de Buenos Aires and Comité de Bioética, Fundación Huésped (Buenos Aires, Argentina). Both HIV-infected participants and HD provided written informed consents accepting to participate in this study in accordance with the Declaration of Helsinki.

## Author Contributions

YG and GT conceived the study and designed the experiments; CT, JS, MR, and YG performed experiments; LL and RB contributed with reagents; CT, RB, MQ, HS, YG, and GT analyzed and interpreted the data; CT and GT wrote the manuscript. All authors read and approved the final version of this manuscript.

## Conflict of Interest Statement

The authors declare that the research was conducted in the absence of any commercial or financial relationships that could be construed as a potential conflict of interest.

## Supplementary Material

Figure S1 Gating strategy used for flow cytometry analysis of monocyte-derived macrophages (MDMs) **(A)** and CD4^+^ T-cells **(B)**. First, doublets were excluded in a forward scatter (FSC)-height (FSC-H) versus an FSH-area (FSH-A) plot. Then, living cells were gated an FSC-A versus a side scatter (SSC) plot. Subsequently, infected cells were identified in an FSC-H versus FITC plot (MDMs) or versus GFP plot (CD4^+^ T-cells). Data acquisition was performed in a BD FACSCanto flow cytometer using the BD FACSDiva software and analyzed subsequently with FlowJO v10 software (Data Analysis Software, LLC).
