# Supplementary figures and images for: Interaction Between Macrophage Migration Inhibitory Factor and CD74 in Human Immunodeficiency Virus Type I Infected Primary Monocyte-Derived Macrophages Triggers the Production of Proinflammatory Mediators and Enhances Infection of Unactivated CD4+ T Cells
Source: Front Immunol. 2018 Jun 27;9:1494. doi: 10.3389/fimmu.2018.01494 (PMC6030361; doi:10.3389/fimmu.2018.01494)

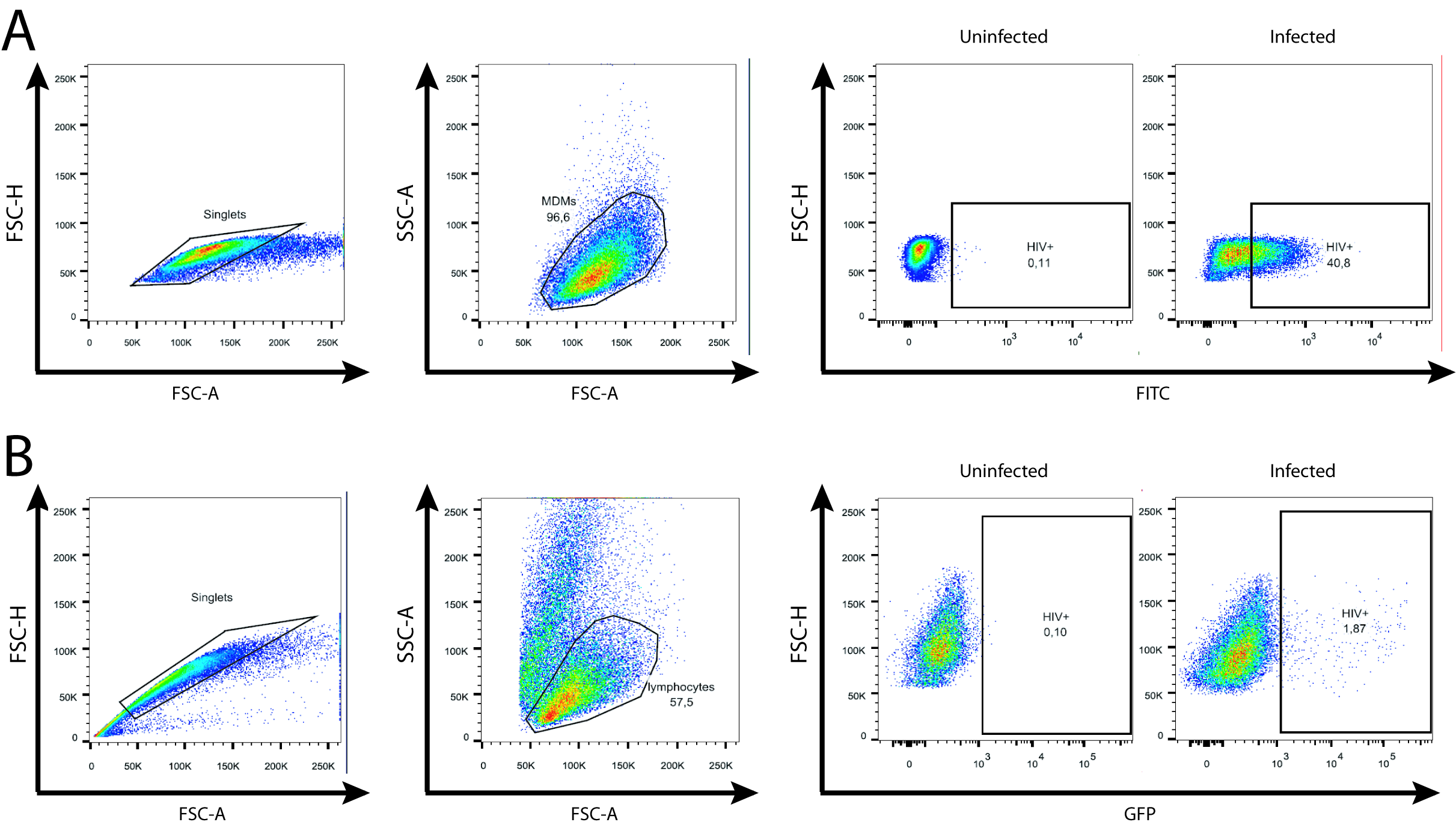

Supplement: Figure S1 — Gating strategy used for flow cytometry analysis of monocyte-derived macrophages (MDMs) (A) and CD4+ T-cells (B). First, doublets were excluded in a forward scatter (FSC)-height (FSC-H) versus an FSH-area (FSH-A) plot. Then, living cells were gated an FSC-A versus a side scatter (SSC) plot. Subsequently, infected cells were identified in an FSC-H versus FITC plot (MDMs) or versus GFP plot (CD4+ T-cells). Data acquisition was performed in a BD FACSCanto flow cytometer using the BD FACSDiva software and analyzed subsequently with FlowJO v10 software (Data Analysis Software, LLC). [file Image_1.tif]
